# Optimizing antiretroviral therapy for children living with HIV: Experience from an observational cohort in Lesotho

**DOI:** 10.1371/journal.pone.0288619

**Published:** 2023-07-17

**Authors:** Vincent J. Tukei, Nicole Herrera, Matseliso Masitha, Lieketseng Masenyetse, Majoalane Mokone, Mafusi Mokone, Limpho Maile, Michelle M. Gill

**Affiliations:** 1 Elizabeth Glaser Pediatric AIDS Foundation, Maseru, Lesotho; 2 Elizabeth Glaser Pediatric AIDS Foundation, Washington, D.C., United States of America; 3 Ministry of Health, Maseru, Lesotho; Kasetsart University, THAILAND

## Abstract

**Introduction:**

We describe transition of HIV-positive children from efavirenz- or nevirapine-based antiretroviral therapy (ART) to optimal dolutegravir (DTG) or lopinavir/ritonavir (LPV/r) (solid formulation)-based ART in Lesotho.

**Methods:**

We followed a cohort of children less than 15 years of age who were initiated on ART on or after January 1, 2018 from 21 selected health facilities in Lesotho. From March 2020 to May 2022, we collected data retrospectively through chart abstraction and prospectively through caregiver interviews to cover a period of 24 months following treatment initiation. We used a structured questionnaire to collect data on demographics, ART regimen, drug formulations and switches, viral suppression, retention, and drug administration challenges. Data were summarized as frequencies and percentages, using SAS ver.9.4.

**Results:**

Of 310 children enrolled in the study, 169 (54.5%) were female, and median age at ART initiation was 5.9 years (IQR 1.1–11.1). During follow-up, 19 (6.1%) children died, 41 (13.2%) were lost to follow-up and 74 (23.9%) transferred to non-study sites. At baseline, 144 (46.4%) children were receiving efavirenz-based ART regimen, 133 (42.9%) LPV/r, 27 (8.7%) DTG, 5 (1.6%) nevirapine; 1 child had incomplete records. By study end, 143 (46.1%) children were receiving LPV/r-based ART regimen, 109 (35.2%) DTG, and 58 (18.7%) were on efavirenz or nevirapine-based regimen. Of 116 children with viral load results after six months or more on a consistent regimen, viral suppression was seen in 35/53 (66.0%) children on LPV/r, 36/38 (94.7%) children on DTG and 19/24 (79.2%) children on efavirenz.

**Conclusion:**

Following optimal ART introduction in Lesotho, most children in the cohort were transitioned and many attained or maintained viral suppression after transition; however, we recommend more robust viral load monitoring and patient tracking to reduce losses and improve outcomes after ART transition.

## Introduction

An estimated 1.7 million children are living with HIV worldwide [1]. A large proportion of these children reside in sub-Saharan Africa. HIV antiretroviral treatment (ART) programs in many sub-Saharan African countries are routinely aligned to World Health Organization (WHO) guidelines. WHO guidelines for HIV treatment have evolved over the years to include more efficacious treatment options as new agents become available. The scarcity of optimal, age-appropriate treatment options for children living with HIV (CLHIV) remains a challenge [2, 3]. Previous WHO guidelines recommended the use of Abacavir (ABC) or Zidovudine (AZT) in combination with Lamivudine (3TC) as the nucleoside reverse transcriptase inhibitor (NRTI) backbone of choice for children together with a third agent that included either a non-nucleoside reverse transcriptase inhibitor (NNRTI) or a boosted protease inhibitor (PI) [4]. Efavirenz (EFV) was the NNRTI of first choice for children 3 years or older, and Lopinavir/ritonavir (LPV/r) was the recommended PI for children less than 3 years. Nevirapine (NVP) was reserved for prophylaxis to prevent mother-to-child-transmission (PMTCT), and as a preferred alternative treatment in situations where EFV or LPV/r were contraindicated or unavailable [4].

The extensive use of NVP for PMTCT and for treatment, has over the decades, led to a build-up of high-level HIV resistance to NNRTI agents [5–10]. Early treatment failure has been reported in children receiving NVP-based ART [11, 12]. Additional studies have shown the superiority of LPV/r over NVP for pediatric HIV treatment [13–15]. Over the last decade, there has been a move away from the use of NVP and EFV-based ART in favor of LPV/r for treatment of CLHIV. However; the administration of LPV/r to infants and young children is complicated by the unpalatability of the liquid formulation, the high alcohol and propylene glycol excipient content in the liquid formulation, and transportation and storage difficulties for the bottles [16]. A heat-stable tablet formulation is available, but it is designed to be swallowed whole, and must not be chewed, broken or crushed, making it difficult to administer to infants and young children who may not need the full tablet dose. In recent years, new pellet and dispersible granule formulations have been approved for use among CLHIV. These alternative formulations provide better options, and efficacious alternatives in settings where NNRTI resistance is expected. However, information on long-term acceptability and challenges of administering these formulations in public health settings remains scanty. In clinical trial settings, acceptability of LPV/r pellets decreased over time [17]. Challenges experienced by caregivers who have to administer these formulations to children for long periods have not been fully documented.

Dolutegravir (DTG), an integrase strand transfer inhibitor (InSTI) was initially approved for use in adults and children 6 years or older who weighed ≥ 20kg. More recently, WHO approved its use in infants and children from 4 weeks of age and a body weight ≥ 3kg [18]. DTG is considered a better alternative to LPV/r and has a superior viral efficacy compared to NNRTIs [19, 20].

In Lesotho, the 2016 national ART guidelines were aligned to WHO guidance that recommended use of ABC+3TC+LPV/r or EFV as first-line ART for children, with NVP as an alternative to LPV/r in young children on treatment for tuberculosis or as an alternative to EFV in older children who did not tolerate EFV [21]. In 2019, ABC+3TC+DTG was introduced as the first-line option for children 3–9 years of age weighing ≥20kg; and tenofovir (TDF)+3TC+DTG was preferred for children ≥10 years. With the introduction of DTG (50mg) and LPV/r pellets and granules, the Ministry of Health (MOH) adopted a phased approach to transition eligible CLHIV from NVP- and EFV-based regimens to either DTG or LPV/r (pellet or granule)-based regimen, depending on age and weight, starting in August 2019. Virologic and clinical outcomes of children transitioned to optimal ART containing DTG or LPV/r solid formulations in the public healthcare setting of Lesotho remain unknown. This study aimed to describe the transition to optimal pediatric ART in Lesotho; assess viral suppression among CLHIV after the transition; and document challenges encountered by caregivers as they administer new formulations to CLHIV.

## Methods

### Design

**Fig 1:**
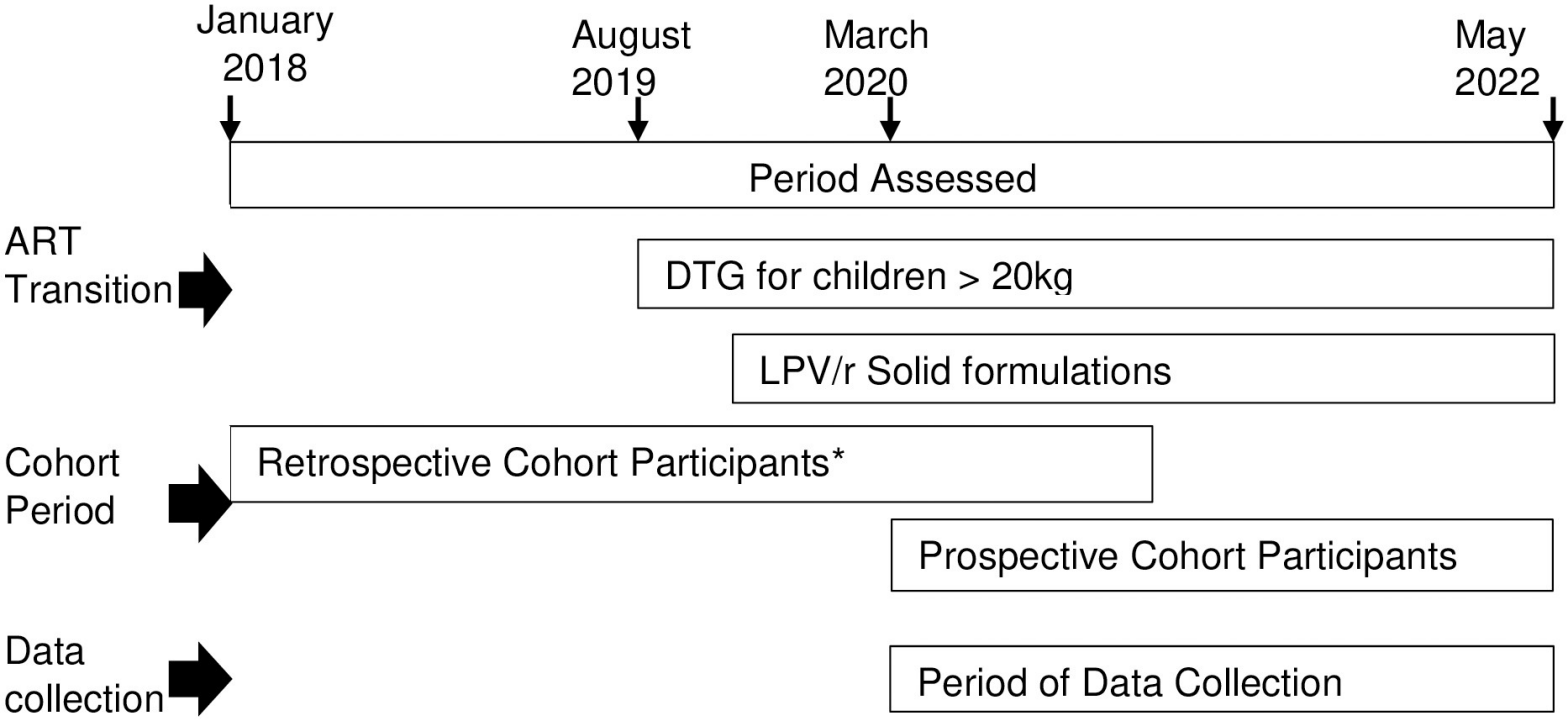
Study design showing period of cohort data collection and ART transition in 21 health facilities in Lesotho; January 2018–May 2022. * Includes all cohort participants who initiated antiretroviral therapy between January 2018 and March 2020.

We enrolled and followed a cohort of HIV-positive children and adolescents 0–19 years of age who were either ART-naïve or started ART on or after January 1, 2018 and received HIV care and treatment from selected study health facilities in Lesotho. This analysis is limited to data from children and adolescents less than 15 years of age. Cohort data were collected prospectively and retrospectively from March 2020 to May 2022. We used a structured questionnaire to collect quantitative data from study participants every 3 months during regular clinic visits until 24 months of ART follow-up. For participants who initiated ART prior to March 2020, data were collected retrospectively to ensure coverage of a period of 24 months from ART initiation. If the participant’s duration on ART was less than 24 months and the participant was still in care, 3-monthly visit data were collected prospectively until 24-month follow-up data were obtained. Retrospective data were obtained through chart abstraction, while prospective follow-up involved participant and/or caregiver interviews as well as abstraction of relevant information from medical records (Fig 1).

### Setting

The study was carried out in 4 districts where the Elizabeth Glaser Pediatric AIDS Foundation (EGPAF) is implementing a United States Agency for International Development (USAID)—funded HIV program for care and treatment of children and adults living with HIV. Program data were used to identify high-volume health facilities that had, by March 2019, registered at least 30 children on ART. In total, 21 high volume health facilities that included all 7 hospitals within the districts and 14 high-volume health centers were selected. At the health facilities, routine HIV care is provided based on WHO “treat-all” approach. Trained counselors provide age-appropriate HIV testing, clinicians prescribe and dispense ART based on national guidelines, and return visits for clinic check-up are scheduled every 3 months. Patients who do not return to the clinics for their scheduled appointments are tracked through phone calls on the day of the missed appointment, and if unavailable, a home visit is scheduled in collaboration with village health workers in charge of the patient’s community. Viral load monitoring for children on ART is carried out every 6 months.

### ART transition during the study period

Following the national policy change on optimized ART regimen in August 2019, health facilities transitioned children (≥ 20kg) from EFV or NVP-based ART to DTG (50mg). Younger children weighing < 20kg, were transitioned to LPV/r (40/10mg) pellets or granules. Infants on liquid LPV/r formulation were transitioned to LPV/r granules. All ART transitions were in response to policy change and were not based on virologic outcomes.

### Participants

The study participants were drawn from a population of children receiving HIV care and treatment services at the study health facilities. Participants were enrolled if they were less than 19 years of age, were confirmed to be HIV-positive based on age-appropriate WHO-approved HIV tests, and initiated ART on or after January 1, 2018. For this analysis, only a subset of children less than 15 years was included.

### Enrolment and follow-up procedures

We consecutively enrolled eligible children during regular clinic visits and interviewed their caregivers to obtain demographic and medical data. We used electronic tablets with built-in structured questionnaires to collect quantitative data on specified study variables. The data were subsequently uploaded to a secure study database for cleaning and analysis. Participant medical records were reviewed to obtain relevant medical history. History and visit data for children retrospectively enrolled because they were no longer in care or had already reached 24 months on ART prior to the time of data collection were abstracted from clinical records. Follow-up data for prospective participants were collected during clinic visits. Data collection was carried out by trained study nurses stationed at the study sites. The nurses conducted caregiver interviews, reviewed patient medical records and extracted data on specified study variables. Caregivers were interviewed together with their children whenever they returned to the clinics for their scheduled visits.

### Variables and data sources

Data on participant demographics, ART regimen, drug formulations, and ART switches were collected. In addition, information on comorbidities, viral suppression following ART initiation, retention in care, and adverse events was obtained. Among participants followed prospectively, primary caregivers were interviewed and a structured questionnaire was used to obtain information on challenges experienced by children and their caregivers during ART drug administration. A primary caregiver was defined as any adult who assumed the most responsibility in caring for the health and well-being of the child/adolescent. To assess participant outcomes, optimal ART formulations were considered the exposure variable, and were defined as ART with a backbone of 2 NRTIs plus either DTG or solid formulations of LPV/r. Viral suppression was the outcome of interest and was defined at two levels: < 1000 copies/ml, a threshold routinely used for assessing progress towards the Joint United Nations Program on HIV/AIDS (UNAIDS) programmatic targets; and < 50 copies/ml for viral undetectability as described in the WHO 2021 guidelines [18, 22, 23]. Participants were considered retained in care if they returned to the health facilities for their scheduled visits. Participants who did not return for their clinic appointments and could not be traced by the end of the 24-month follow-up period were considered lost to follow-up at the visit where they were last seen.

### Statistical analysis

Baseline categorical demographic, social and clinical characteristics were described as frequencies and percentages while continuous variables were summarized as medians with interquartile ranges. Viral suppression and retention outcomes were summarized using proportions. All data were analysed with SAS software version 9.4 (SAS Institute Inc., Cary, NC, USA).

### Ethics

Ethical clearance to conduct the study was obtained from the Lesotho Ministry of Health’s Research Ethics Committee (IRB00009860) and the Advarra Institutional Review Board (IRB) (IRB00000971) in the United States of America. Caregivers provided written informed consent. In addition, verbal assent was obtained from children 12 years or older. A waiver of consent was obtained from the IRB to abstract data from medical records of patients who had been on ART for 24 months or lost to-follow-up at the time of data collection and were enrolled retrospectively. To maintain confidentiality, participants were assigned unique study identification numbers that were used throughout the data collection process and analysis. Before analysis, all data were anonymized and no information linking to participants were collected. Authors did not have access to identifiable data.

### Inclusivity in global research

This research was carried out by the Elizabeth Glaser Pediatric AIDS Foundation in close collaboration with the Lesotho Ministry of Health. Additional information regarding the ethical, cultural, and scientific considerations specific to inclusivity in global research is included in the Supporting Information (S1 Checklist).

## Results

### Demographic characteristics

A total of 310 children <15 years were enrolled in the study; 236 (76.1%) were enrolled retrospectively (started ART before the period of data collection), while the remainder were prospectively enrolled. The median age at ART initiation was 5.9 years (IQR 1.1–11.1), and 144 (46.4%) children were below 5 years of age (Table 1). At ART initiation, 144 (46.4%) children were initiated on an EFV-based regimen, 133 (42.9%) on LPV/r, 5 (1.6%) on NVP and 27 (8.7%) on DTG-based regimen. One hundred twenty-one (84.0%) of 144 children on EFV; 4 (80.0%) of 5 on NVP; 94 (70.7%) of 133 on LPV/r; and 16 (59.3%) of 27 on DTG started treatment before the period of data collection. Of those who initiated LPV/r; 11 (8.3%) were on tablets, 102 (77.3%) were on liquid formulation, 19 (14.4%) were on pellets, and 1 did not have a formulation specified. One child on ABC+3TC did not have record of the third drug.

**Table 1:** Demographic and treatment characteristics of 310 children (0–14 years of age) at ART initiation in 21 health facilities in Lesotho; January 2018-May 2022.

Characteristic	N (%)
Age (Years) at ART initiation	
< 5	144 (46.4)
5–9	78 (25.2)
10–14	88 (28.4)
Sex	
Female	169 (54.5)
District	
Maseru	163 (52.6)
Mafeteng	72 (23.2)
Mohale’s Hoek	39 (12.6)
Thaba Tseka	36 (11.6)
Type of enrolment	
Prospective	74 (23.9)
Retrospective	236 (76.1)
ART regimen at treatment initiation	
ABC+3TC+LPV/r	132 (42.6)
ABC+3TC+EFV	104 (33.6)
TDF+3TC+EFV	32 (10.3)
ABC+3TC+DTG	15 (4.8)
Other	27 (8.7)
HIV+ status disclosed to child*	22/33 (66.7)

ART = Antiretroviral therapy; ABC = abacavir; 3TC = lamivudine; LPV/r = lopinavir/ritonavir; EFV = efavirenz; DTG = dolutegravir

*Question only asked as part of the prospective cohort and reported on those > 5 years of age.

### Cohort follow-up

**Fig 2:**
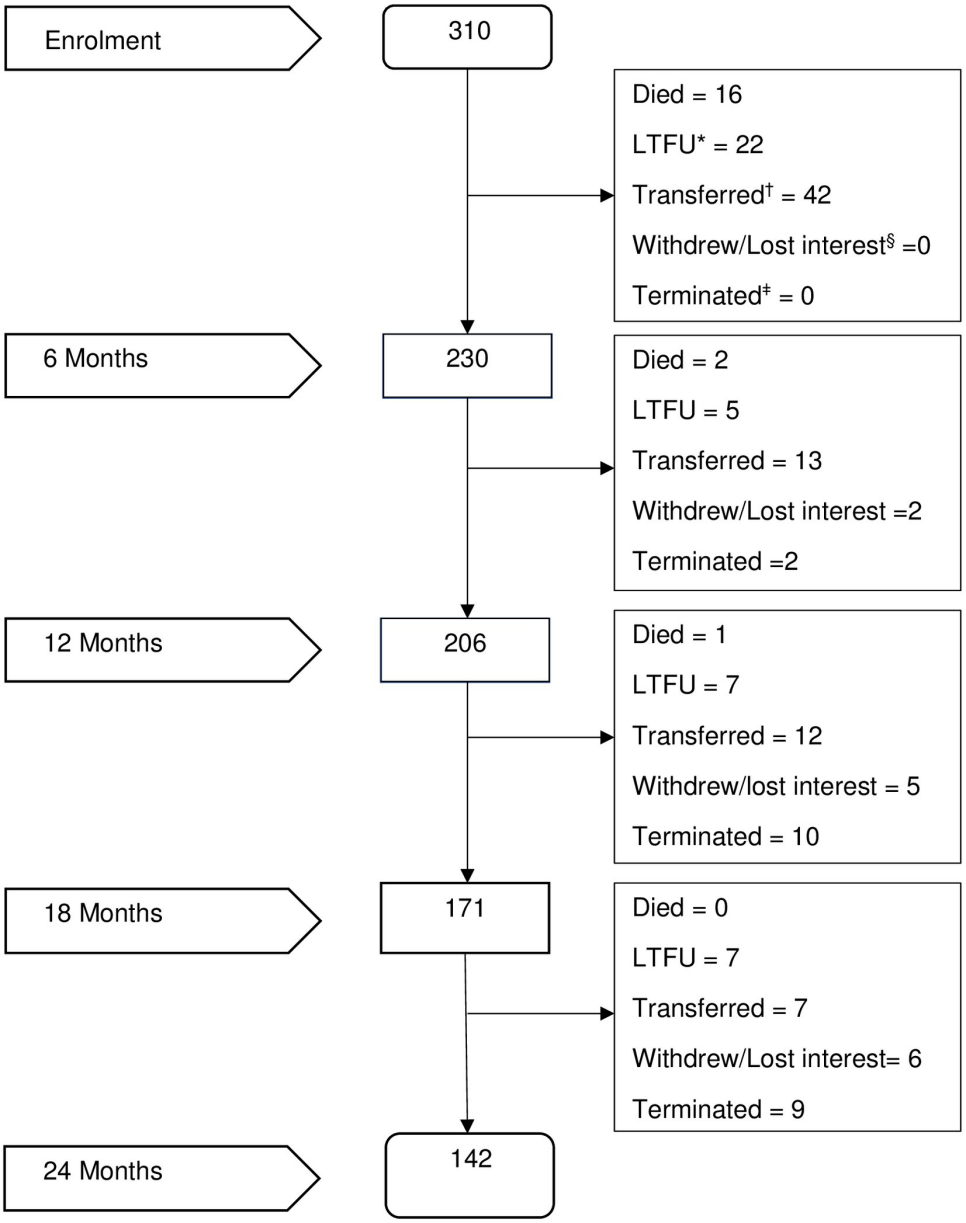
Follow-up outcomes of 310 participants enrolled in 21 study health facilities; January 2018–May 2022. *LTFU = Loss to follow-up; ^†^Transferred = Participant relocated to non-study site. ^§^Withdrew/Lost interest: participant remained in care but either withdrew from the study or were no longer interested in study follow-up. ^ǂ^Terminated = Due to resource constraints, the study was stopped before some participants completed 24 months follow-up.

During the study period, a total of 248 (80.0%) participants were followed for a median duration of 23.2 months (IQR: 14.7–24.3). Sixty-two (20.0%) participants did not return after the baseline visit. Nineteen (6.1%) children died during follow-up, 41 (13.2%) were lost to follow-up and 74 (23.9%) transferred to non-study sites. Of those who died, 17 (89.5%) were under 5 years of age, and 2 (10.5%) were 5–9 years of age. Sixteen (84.2%) of the 19 deaths occurred within the first 6 months of ART initiation (Fig 2). Twenty-one participants who entered the cohort late did not complete the 24 months and were terminated due to resource constraints that led to early study closure, contributing a median of 14.9 (interquartile range: 13.3–17.6) months of study follow-up. Of the 62 who only had a baseline visit, 57 (91.1%) were enrolled retrospectively. The proportion of participants who did not return after enrolment was higher among retrospective participants, 57/236 (24.2%) compared to prospectively enrolled participants, 5/74 (6.8%); p = 0.001. Of those considered lost to follow-up at baseline, 40 (27.8%) were children under 5 years of age; 12 (15.4%) were 5–9 years and 10 (11.4%) were adolescents, 10–14 years.

### Antiretroviral therapy during follow-up

All children were initiated on ART. Follow-up data were available for the 248 children who returned to the clinics after the enrolment visit. Of these, 147 (59.3%) children never changed ART regimens, 97 (39.1%) had one regimen change, three (1.2%) had two changes within the study period, and one (0.4%) child initially seen at baseline, had only one return visit to the clinic at 15 months and was considered lost to follow-up. Of the children whose ART regimen was not changed, 88 (59.9%) were on LPV/r, 37 (25.2%) were on EFV, 20 (13.6%) were on DTG and 2 (1.4%) were on NVP. Of those who were on LPV/r throughout follow-up, 34 (38.6%) did not switch formulations; 3 on tablets, 26 on syrup, and 5 on pellets.

Ninety-three (95.9%) of 97 children with a single regimen change, were transitioned from an NNRTI-based regimen to optimal regimen containing either LPV/r (solid formulation) or DTG. Two children initially on solid (one on tablets, one on pellets) LPV/r were switched to DTG; one child was changed from LPV/r to EFV and the other child switched from NVP to EFV (Table 2).

**Table 2:** ART switches and substitutions by age category documented at 21 health facilities in Lesotho, January 2018–May 2022.

N (%)	Children <5 years (N = 144)	Children 5–9 years (N = 78)	Adolescents 10–14 years (N = 88)	Total (N = 310)
**Regimen switches across follow-up**	103	66	78	247
Zero switches	92 (89.3)	20 (30.3)	35 (44.9)	147 (59.5)
One switch	10 (9.7)	45 (68.2)	42 (53.9)	97 (39.3)
Two switches	1 (1.0)	1 (1.5)	1 (1.3)	3 (1.2)
**Children with one switch**	10	45	42	97
NVP to LPV/r	3 (30.0)	-	-	3 (3.1)
EFV to LPV/r	6 (60.0)	5 (11.1)	1 (2.4)	12 (12.4)
LPV/r to EFV	1 (10.0)	-	-	1 (1.0)
LPV/r to DTG	-	2 (4.4)	-	2 (2.1)
EFV to DTG	-	37 (82.2)	41 (97.6)	78 (80.4)
NVP to EFV	-	1 (2.2)	-	1 (1.0)
**Children with two switches**	1	1	1	3
EFV to LPV/r to DTG	-	1	-	1
EFV to NVP to DTG	-	-	1	1
LPV/r to NVP to DTG	1	-	-	1

ART = Antiretroviral therapy; NNRTI = non-nucleoside reverse transcriptase inhibitor; PI = protease inhibitor; NVP = nevirapine; LPV/r = lopinavir/ritonavir; EFV = efavirenz; InSTI = integrase strand inhibitor; DTG = dolutegravir

At the end of study follow-up, 143 (46.1%) children were on LPV/r, 109 (35.2%) were on DTG and 58 (18.7%) were on non-optimal (NNRTI) ART regimen. Of the 143 on LPV/r, 24 (16.8%) were on tablets, 53 (37.1%) were on liquid formulation, 55 (38.5%) were on pellets, and 11 (7.7%) were on granules.

### Transition to LPV/r solid formulations

A total of 148 children received LPV/r at some point during follow-up. Of these, 90 (60.8%) children remained on a single formulation, 48 (32.4%) had a single change in LPV/r formulation, eight (5.4%) changed LPV/r formulations twice, and two (1.4%) children changed LPV/r formulations three times. Of the 90 children who remained on a single LPV/r formulation, 20 (22.2%) were on tablets, 54 (60%) were on liquid formulation and 16 (17.8%) were on pellets. Fifty three of 54 participants on liquid LPV/r formulation were enrolled retrospectively (treatment initiated before March 2020), with 27 (50%) of them having received LPV/r on a single visit and were thereafter lost to follow-up. Most of the LPV/r formulation changes were seen in the under 5 age group. Forty-one (85.4%) of 48 participants with a single LPV/r formulation change and eight (80%) of ten children with two or more LPV/r formulation changes were switched from liquid to solid formulations (Table 3).

**Table 3:** Changes in LPV/r formulations by type of enrolment at 21 health facilities in Lesotho; January 2018–ay 2022.

LPV/r Formulation switch N (%)	Total (N = 58)	Children under 5 years (N = 55)	Children 5–9 years (N = 3)
**One formulation switch**	**48**	**46**	**2**
Syrup to pellets	35 (72.9)	35 (76.1)	0 (0)
Syrup to tablet	5 (10.4)	4 (8.7)	1 (50.0)
Syrup to granules	1 (2.1)	1 (2.2)	0 (0)
Tablet to pellet	2 (4.2)	1 (2.2)	1 (50.0)
Pellet to granules	5 (10.4)	5 (10.9)	0 (0)
**Two formulation switches**	**8**	**7**	**1**
Syrup-pellet-granules	4	4 (57.1)	0 (0)
Syrup-tablet-syrup	1	1 (14.3)	0 (0)
Syrup-tablet-pellet	1	1 (14.3)	0 (0)
Pellet-tablet-granules	1	1 (14.3)	0 (0)
Pellet-granules-tablet	1	0	1 (100.0)
**Three formulation switches**	**2**	**2**	**0**
Syrup-granules-syrup-tablet	1 (50.0)	1 (50.0)	0
Syrup-tablet-syrup-tablet	1 (50.0)	1 (50.0)	0

LPV/r = lopinavir/ritonavir

### Viral suppression

Viral load results were only available for 189 (61.0%) children. To evaluate viral load suppression (VLS) we isolated the analysis to focus on those who had at least 6 months of follow-up data (N = 180). Of these, 58 (32.2%) were children < 5 years of age, 55 (30.6%) were 5–9 years and 67 (37.2%) were adolescents 10–14 years. When evaluating the final viral load result amongst the 180 who had at least 6 months of follow up data, a total of 148 (82.2%) children attained viral suppression; 119 (66.1%) had undetectable viral loads, and 29 (16.1%) had viral loads between 50–999 copies/ml.

When evaluating viral suppression across age groups, we considered those who had been on the same ART regimen for at least 6 months and had a viral load test done at least 6 months after initiation an ART regimen. This was done to best reflect a viral load associated with a regimen of interest; there were 116 children who met this criterion. Among children < 5 years of age who had been on ART for a median time of 13.4 months (IQR: 9.8–19.8), 40 (69.0%) children attained viral suppression, of which 25 (43.1%) had undetectable viral load. Among 5-9-year-olds on ART for a median time of 19.9 months (IQR: 16.3–23.0), 49 (89.1%) attained viral suppression, with 45 (81.8%) of them having undetectable viral load. The median ART duration for adolescents 10–14 years was 17.9 months (IQR: 11.4–22.2). Fifty-nine (88.1%) adolescents attained viral suppression, with 49 (73.1%) of them having undetectable viral load.

Of these, 53 (45.7%) were on LPV/r, 38 (32.8%) were on DTG, 24 (20.7%) were on EFV and 1 (0.8%) was on NVP. Viral suppression was attained in 35 (66.0%) children on LPV/r, 36 (94.7%) children on DTG, and 19 (79.2%) children on EFV. Of the 35 children on LPV/r who achieved suppression, only 8 (22.9%) did not switch formulations, 24 (68.6%) had one formulation switch (23 from syrup to pellets or tablets, and 1 from pellet to granules), and 3 (8.6%) had two formulation switches (2 from syrup to pellet to granule; and 1 from syrup to tablet to granules). The one child on NVP did not attain viral suppression (Table 4).

**Table 4:** Viral suppression by age and drug regimen, among 116 children with VL result after ≥6 months of consecutive drug: Results from 21 health facilities in Lesotho; January 2018-May 2022.

Variable	Age Category (years)			Total (n = 116)
	< 5 (n = 51)	5–9 (n = 24)	10–14 (n = 41)	
Viral load result n (%)				
Suppressed	35 (68.6)	21 (87.5)	34 (82.9)	90 (77.6)
Unsuppressed	16 (31.4)	3 (12.5)	7 (17.1)	26 (22.4)
Median time on regimen at viral load report; months (interquartile range)	13.1 (8.9–18.9)	10.0 (8.0–15.5)	15.0 (9.0–18.4)	13.2 (8.7–18.1)
Viral load result after ≥ 6 months on DTG; n (%)	-	(n = 12)	(n = 26)	(n = 38)
Suppressed	-	12 (100.0)	24 (92.3)	36 (94.7)
Unsuppressed	-	-	2 (7.7)	2 (5.3)
Median time (months) on DTG		10.0 (7.3–15.9)	12.6 (8.2–15.6)	11.6 (7.8–15.9)
Viral load after ≥ 6 months on LPV/r; n (%)	(n = 46)	(n = 7)	-	(n = 53)
Suppressed	31 (67.4)	4 (57.1)	-	35 (66.0)
Unsuppressed	15 (32.6)	3 (42.9)	-	18 (34.0)
Median time (months) on LPV/r	13.0 (7.4–15.2)	13.5 (7.4–15.2)	-	13.1 (8.9–18.9)
Viral load results after ≥ 6 months on EFV; n (%)	(n = 4)	(n = 5)	(n = 15)	(n = 24)
Suppressed	4 (100)	5 (100)	10 (66.7)	19 (79.2)
Unsuppressed	-	-	5 (33.3)	5 (20.8)
Median time (months) on EFV	16.9 (11.5–20.9)	9.7 (8.4–13.5)	18.2 (11.4–20.6)	17.3 (9.8–20.4)
Viral load results after ≥ 6 months on NVP; n (%)	(n = 1)	-	-	(n = 1)
Suppressed	-	-	-	-
Unsuppressed	1 (100.0)	-	-	1 (100)
Median time (months) on NVP	10.4 (10.4–10.4)	-	-	10.4 (10.4–10.4)

Of 53 children who were on LPV/r for ≥ 6 months and had viral load results, viral suppression at the end of follow-up was attained in 8 (80%) of 10 children who transitioned to tablets, 21 (70%) of 30 children transitioned to pellets, 3 (37.5%) of 8 children transitioned to granules and in 3 (60%) of 5 children who were still on liquid formulation at the last visit.

Viral load results were available for 29 participants with a sample collected between 3 and 9 months following consecutive DTG ART use (as a proxy for 6-month viral load) and 19 participants with a sample collected between 9 and 15 months following consecutive DTG ART use (as a proxy for 12-month viral load). Among children with an approximate 6-month viral load result, 25 (86.2%) attained viral suppression, of which 21 had an undetectable viral load. Viral suppression was attained in 10/11 children who were newly initiated on DTG and in 15/18 children who switched to DTG from another regimen. All 19 children with an approximate 12-month viral load result had an undetectable viral load. Of these children, 7 were newly initiated and 12 switched to DTG from another regimen.

### Challenges reported by caregivers

Altogether 74 caregivers of prospectively enrolled participants were interviewed on challenges related to ART administration. Ten caregivers reported difficulties in administering medication to their children at one (n = 4) or multiple visits (n = 6). All the difficulties reported were for children < 5 years receiving ABC+3TC+LPV/r. Among the 9 who reported spitting or vomiting after taking LPV/r, 1 was on syrup, 1 was on pellets/granules, 5 were on tablets, and 2 had an unknown formulation. One caregiver reported the difficulty was that drugs had to be administered at an inconvenient time.

## Discussion

Our study, conducted in the real-world public health setting of Lesotho, described transition of an ART program for CLHIV, from suboptimal NNRTI-based or liquid LPV/r-based regimens to optimal DTG or solid LPV/r-based formulations that are known to improve participant outcomes. Nearly half of the participants started with an NNRTI-based regimen, and by study end, 81.3% of children were on optimal LPV/r or DTG regimen. Overall, 82.2% of participants attained viral suppression with a notably higher proportion (94.7%) of viral suppression seen among children who transitioned to DTG.

Viral resistance to NVP and NNRTIs in general, has been widely known for the last two decades [6, 24–26], and yet, as shown in this cohort, nearly half of the children were initiated on EFV and NVP and continued to depend on these suboptimal medications in the absence of more effective child-appropriate options. ART optimization for children has traditionally lagged behind that of adults partly because evidence on the effectiveness and safety of new agents often comes from trials that exclude children; however, even when the evidence is available, development of appropriate formulations for children and ensuring their availability in places where they are needed often comes with additional delays. For example, the LPV/r pellets used in this cohort, were approved by the United States Food and Drug Administration (US FDA) in 2015 but only became available to the children in our cohort 4 to 5 years later [27].

Despite the delays, many of the children were able to attain and maintain viral suppression. The high viral suppression seen in children who transitioned to DTG aligns with results from clinical trials and other adult cohorts elsewhere [28–31]. We did not collect data on viral loads before transition, but it is likely that viral suppression was attained even among children who may have had an unsuppressed viral load at the time of transition. These findings reinforce the WHO guidance for use of DTG for first and second-line treatment of CLHIV [18].

The virologic response in children on LPV/r was, however, less impressive. We note that there were marked age differences between patients on DTG and those on LPV/r, making it difficult for any direct comparisons to be made; however, it is worth noting that only two thirds of children on LPV/r attained viral suppression. The majority of children on LPV/r were under 5 years of age and had transitioned from liquid to solid formulations. We cannot fully explain the poor performance of LPV/r; however, difficulties in administration of LPV/r liquid formulation, previously observed in other cohorts [17, 32], may have led to subtherapeutic dosing in some children prior to the transition to solid formulation. The relatively low performance of LPV/r could also be reflective of waning acceptability of pellets as reported by others [17]. LPV/r is known to have a high genetic barrier to viral resistance [33]; however, prolonged subtherapeutic dosing may lead to an accumulation of resistant viral strains. Though resistance testing was not carried out, the lack of viral suppression after transitioning to solid formulations suggests possible resistance to LPV/r. We expect to see virological outcome improvements in this group of children following the roll out of the pediatric DTG (10mg) formulation in Lesotho in April 2022.

Some children on LPV/r were started on a liquid formulation, transitioned to solid formulation, and later reverted to liquid formulations. These many changes may cause confusion among caregivers which may result in suboptimal dosing of ART. We did not collect data to explain these frequent changes; however, in settings where temporary stock-outs of optimal formulations occur, it is not uncommon for clinicians to prescribe the available formulation until the appropriate formulation is re-stocked.

Changing ART often introduces new challenges since caregivers have to learn to administer the new drugs or formulations and look out for any challenges or side effects that may arise. Among caregivers who were interviewed on drug administration challenges, only a small minority (13.5%) reported difficulties in administering medication. This is reassuring and agrees with findings of a similar study conducted in Zimbabwe that interviewed 74 caregivers [34]. In contrast to the Zimbabwe study in which 36% of caregivers reported poor taste as one of the difficulties in administration of LPV/r pellets, none of the caregivers in our cohort reported drug administration difficulties resulting from poor taste.

Over the 2-year period of follow-up, 134 of 310 participants were lost from the cohort. The majority of these children (n = 74, 55%) relocated or transferred to other non-study clinics for their chronic care; others (n = 41) were lost to follow-up, and some (n = 19) died. Patient transfers from one clinic to another is common and has been documented in other cohorts within the region [35, 36]. In settings like Lesotho, where family migration is common [37], optimization of pediatric ART programs should include robust patient follow-up systems that ensure continuity of care whenever transfers occur. Such systems should have mechanisms for tracking lost patients and ensuring their reengagement into care.

The high mortality seen within the first 6 months of ART, particularly among young children, has been documented by others [38, 39]. It reinforces the need for early initiation of efficacious ART in age-appropriate formulations, coupled with the need for close monitoring and follow-up to ensure continuity of care for this vulnerable group of children.

Our study had several limitations. First, we largely relied on extraction of data that were routinely collected for patient care in public health settings, particularly as three-quarters of our cohort were enrolled retrospectively. The analysis was therefore limited to variables available in the routine records and some of the source documents had incomplete data for key study variables. Secondly, our cohort had suboptimal viral load coverage and we did not conduct resistance testing for participants who did not achieve viral suppression. We were therefore, not able to definitively determine whether the persistent viremia was due to poor adherence to medication or resistance to ART. Thirdly, we were unable to determine final outcomes of participants who transferred to non-study facilities. This may have led to an underestimation of mortality and loss to follow-up. Despite these limitations, our study provides evidence that is relevant for design and implementation of an effective ART transition program in similar settings. In addition, the results provide real-world outcomes of pediatric ART optimization and associated challenges such as difficulty in maintaining continuity of care, frequent formulation switches, and inadequate viral load monitoring, that often occur in settings outside of clinical trials.

## Conclusion

Our study demonstrated that following change of the national ART policy in Lesotho, many children who received suboptimal ART in the public health setting were successfully transitioned to optimal LPV/r solid or DTG-based ART regimen; and many of the children maintained or attained viral suppression after transition. However, inadequate viral load monitoring, frequent formulation changes, particularly for children who were on LPV/r, and poor retention in care were a challenge to the ART program. To adequately monitor patient outcomes after transition to optimal ART regimen, we recommend establishment of a patient care system that includes robust viral load monitoring and follow-up.

## Supporting information

S1 File Inclusivity in global research. (DOCX)

S1 Dataset (XLSX)

S1 Checklist STROBE statement—Checklist of items that should be included in reports of observational studies. (DOCX)
