# Supplementary material for: Optimizing antiretroviral therapy for children living with HIV: Experience from an observational cohort in Lesotho
Source: PLoS One. 2023 Jul 17;18(7):e0288619. doi: 10.1371/journal.pone.0288619 (PMC10351696; doi:10.1371/journal.pone.0288619)
Supplement: S1 Checklist — (DOCX) [file pone.0288619.s003.docx]

STROBE Statement—checklist of items that should be included in reports of observational studies

|  | Item No. | Recommendation | Page  No. | Relevant text from manuscript |
| --- | --- | --- | --- | --- |
| **Title and abstract** | 1 | (*a*) Indicate the study’s design with a commonly used term in the title or the abstract | Title Page | Observational cohort |
|  |  | (*b*) Provide in the abstract an informative and balanced summary of what was done and what was found | Page 2 |  |
| Introduction | | | |  |
| Background/rationale | 2 | Explain the scientific background and rationale for the investigation being reported | Page 4, line 77-81;  line 95-97 | information on long-term acceptability and challenges of administering these formulations in public health settings remains scanty. In clinical trial settings, acceptability of LPV/r pellets decreased over time (17). Challenges experienced by caregivers who have to administer these formulations to children for long periods have not been fully documented.  Virologic and clinical outcomes of children transitioned to optimal ART containing DTG or LPV/r solid formulations in the public healthcare setting of Lesotho remain unknown. |
| Objectives | 3 | State specific objectives, including any prespecified hypotheses | Page 4, line 97-100 | This study aimed to describe the transition to optimal pediatric ART in Lesotho; assess viral suppression among CLHIV after the transition; and document challenges encountered by caregivers as they administer new formulations to CLHIV. |
| Methods | | | |  |
| Study design | 4 | Present key elements of study design early in the paper | Page 5, line 103-114 | We enrolled and followed a cohort of HIV-positive children less than 15 years of age who were either ART-naïve or started ART on or after January 1, 2018 and received HIV care and treatment from selected study health facilities in Lesotho |
| Setting | 5 | Describe the setting, locations, and relevant dates, including periods of recruitment, exposure, follow-up, and data collection | Page 5, line 115-124 | The study was carried out in 4 districts where the Elizabeth Glaser Pediatric AIDS Foundation (EGPAF) is implementing a United States Agency for International Development (USAID) – funded HIV program for care and treatment of children and adults living with HIV. Program data were used to identify high-volume health facilities that had, by March 2019, registered at least 30 children on ART. In total, 21 high volume health facilities that included all 7 hospitals within the districts and 14 high-volume health centers were selected |
| Participants | 6 | (*a*) *Cohort study*—Give the eligibility criteria, and the sources and methods of selection of participants. Describe methods of follow-up  *Case-control study*—Give the eligibility criteria, and the sources and methods of case ascertainment and control selection. Give the rationale for the choice of cases and controls  *Cross-sectional study*—Give the eligibility criteria, and the sources and methods of selection of participants | Page 5; line 125-129  Page 6; 131-132 | The study participants were drawn from a population of children receiving HIV care and treatment services at the study health facilities. Participants were enrolled if they were less than 19 years of age, were confirmed to be HIV-positive based on age-appropriate WHO-approved HIV tests, and initiated ART on or after January 1, 2018. For this analysis, only a subset of children 0-14 years was included.  We consecutively enrolled eligible children during regular clinic visits and interviewed their caregivers to obtain demographic and medical data |
|  |  | (*b*) *Cohort study*—For matched studies, give matching criteria and number of exposed and unexposed  *Case-control study*—For matched studies, give matching criteria and the number of controls per case |  |  |
| Variables | 7 | Clearly define all outcomes, exposures, predictors, potential confounders, and effect modifiers. Give diagnostic criteria, if applicable | Page 6; line 138-151 |  |
| Data sources/ measurement | 8* | For each variable of interest, give sources of data and details of methods of assessment (measurement). Describe comparability of assessment methods if there is more than one group | Page 6; line 133-137 | Participant medical records were reviewed to obtain relevant medical history. History and visit data for children retrospectively enrolled because they were no longer in care or had already reached 24 months on ART prior to the time of data collection were abstracted from clinical records. Follow-up data for prospective participants were collected during clinic visits. |
| Bias | 9 | Describe any efforts to address potential sources of bias | Page 6; line 131-132 to avoid bias in participant selection | We consecutively enrolled eligible children during regular clinic visits and interviewed their caregivers to obtain demographic and medical data |
| Study size | 10 | Explain how the study size was arrived at | Page 5; line 125-129 | The study participants were drawn from a population of children receiving HIV care and treatment services at the study health facilities. Participants were enrolled if they were less than 19 years of age, were confirmed to be HIV-positive based on age-appropriate WHO-approved HIV tests, and initiated ART on or after January 1, 2018. For this analysis, only a subset of children 0-14 years was included. |

Continued on next page

| Quantitative variables | 11 | Explain how quantitative variables were handled in the analyses. If applicable, describe which groupings were chosen and why | Page 6-7; line 152-154 | Baseline categorical demographic, social and clinical characteristics were described as frequencies and percentages while continuous variables were summarized as medians with interquartile ranges. |
| --- | --- | --- | --- | --- |
| Statistical methods | 12 | (*a*) Describe all statistical methods, including those used to control for confounding | Page 6-7; line 154-157 | Viral suppression and retention outcomes were summarized using proportions and associated 95% confidence intervals. Statistical significance for comparisons was set at 0.05 α level. All data were analysed with SAS version 9.4. |
|  |  | (*b*) Describe any methods used to examine subgroups and interactions | N/A |  |
|  |  | (*c*) Explain how missing data were addressed | N/A |  |
|  |  | (*d*) *Cohort study*—If applicable, explain how loss to follow-up was addressed  *Case-control study*—If applicable, explain how matching of cases and controls was addressed  *Cross-sectional study*—If applicable, describe analytical methods taking account of sampling strategy | N/A |  |
|  |  | (*e*) Describe any sensitivity analyses | N/A |  |
| Results | | | | |
| Participants | 13* | (a) Report numbers of individuals at each stage of study—eg numbers potentially eligible, examined for eligibility, confirmed eligible, included in the study, completing follow-up, and analysed | Fig 1 |  |
|  |  | (b) Give reasons for non-participation at each stage | Fig 1 |  |
|  |  | (c) Consider use of a flow diagram | Fig 1 |  |
| Descriptive data | 14* | (a) Give characteristics of study participants (eg demographic, clinical, social) and information on exposures and potential confounders | Page 7-8; line 169-180 | A total of 310 children <15 years were enrolled in the study; 236 (76.1%) were enrolled retrospectively, while the remainder were prospectively enrolled. The median age at ART initiation was 5.9 years (IQR 1.1-11.1), and 144 (46.4%) children were below 5 years of age (Table 1). At ART initiation, 144 (46.4%) children were initiated on an EFV-based regimen, 133 (42.9%) on LPV/r, 5 (1.6%) on NVP and 27 (8.7%) on DTG-based regimen. Of those who initiated LPV/r; 11 (8.3%) were on tablets, 102 (77.3%) were on liquid formulation, 19 (14.4%) were on pellets, and 1 did not have a formulation specified. One child on ABC+3TC did not have record of the third drug. |
|  |  | (b) Indicate number of participants with missing data for each variable of interest | Page 9; line 198-200  Page 12, line 231-233 | All children were initiated on ART. Follow-up data were available for 248 children who returned to the clinics after the enrolment visit  Viral load results were only available for 189 (61.0%) children during the study period. Among children with at least 6 months of follow-up data, HIV viral load results were available for 180 children. |
|  |  | (c) *Cohort study*—Summarise follow-up time (eg, average and total amount) | Page 8, line 183-185 | During the study period, a total of 248 (80.0%) participants were followed for a median duration of 23.2 months (IQR: 14.7-24.3). Sixty-two (20.0%) participants did not return after the baseline visit. |
| Outcome data | 15* | *Cohort study*—Report numbers of outcome events or summary measures over time | Page 12; line 231-263; Table 4 |  |
|  |  | *Case-control study—*Report numbers in each exposure category, or summary measures of exposure | N/A |  |
|  |  | *Cross-sectional study—*Report numbers of outcome events or summary measures | N/A |  |
| Main results | 16 | (*a*) Give unadjusted estimates and, if applicable, confounder-adjusted estimates and their precision (eg, 95% confidence interval). Make clear which confounders were adjusted for and why they were included | N/A |  |
|  |  | (*b*) Report category boundaries when continuous variables were categorized | Table 1 and Table 2, age categories |  |
|  |  | (*c*) If relevant, consider translating estimates of relative risk into absolute risk for a meaningful time period | N/A |  |

Continued on next page

| Other analyses | 17 | Report other analyses done—eg analyses of subgroups and interactions, and sensitivity analyses | N/A |  |
| --- | --- | --- | --- | --- |
| Discussion | | | | |
| Key results | 18 | Summarise key results with reference to study objectives | Page 16; line 274-260 | Nearly half of the participants started with an NNRTI-based regimen, and by study end, 81.3% of children were on optimal LPV/r or DTG regimen. Overall, 82.2% of participants attained viral suppression with a notably higher proportion (94.7%) of viral suppression seen among children who transitioned to DTG. |
| Limitations | 19 | Discuss limitations of the study, taking into account sources of potential bias or imprecision. Discuss both direction and magnitude of any potential bias | Page 19; line 336-344 | Our study had several limitations. First, we largely relied on extraction of data that were routinely collected for patient care in public health settings, particularly as three-quarters of our cohort were enrolled retrospectively. Some of the source documents had incomplete data for key study variables. Secondly, our cohort had suboptimal viral load coverage and we did not conduct resistance testing for participants who did not achieve viral suppression. We were therefore, not able to definitively determine whether the persistent viremia was due to poor adherence to medication or resistance to ART. Thirdly, we were unable to determine final outcomes of participants who transferred to non-study facilities. This may have led to an underestimation of mortality and loss to follow-up. |
| Interpretation | 20 | Give a cautious overall interpretation of results considering objectives, limitations, multiplicity of analyses, results from similar studies, and other relevant evidence | Page 17-18; line 292-309 |  |
| Generalisability | 21 | Discuss the generalisability (external validity) of the study results | Page 19; line 344-349 | our study provides evidence that is relevant for design and implementation of an effective ART transition program in similar settings. |
| Other information | |  | | |
| Funding | 22 | Give the source of funding and the role of the funders for the present study and, if applicable, for the original study on which the present article is based | Funding statement to be included as part of manuscript submission requirement | The funders had no role in study design, data collection and analysis, decision to publish, or preparation of the manuscript |

*Give information separately for cases and controls in case-control studies and, if applicable, for exposed and unexposed groups in cohort and cross-sectional studies.

**Note:** An Explanation and Elaboration article discusses each checklist item and gives methodological background and published examples of transparent reporting. The STROBE checklist is best used in conjunction with this article (freely available on the Web sites of PLoS Medicine at http://www.plosmedicine.org/, Annals of Internal Medicine at http://www.annals.org/, and Epidemiology at http://www.epidem.com/). Information on the STROBE Initiative is available at www.strobe-statement.org.
